# Robotic monitoring of dunes: a dataset from the EU habitats 2110 and 2120 in Sardinia (Italy)

**DOI:** 10.1038/s41597-024-03063-z

**Published:** 2024-02-24

**Authors:** Franco Angelini, Mathew J. Pollayil, Giovanni Rivieccio, Maria Carmela Caria, Simonetta Bagella, Manolo Garabini

**Affiliations:** 1 Centro di Ricerca “Enrico Piaggio”, and Dipartimento di Ingegneria dell’Informazione, Università di Pisa, Largo Lucio Lazzarino 1, 56122 Pisa, Italy; 2 University of Sassari, Department of Chemistry and Physical, Mathematical and Natural Sciences, Via Vienna 2, I-07100 Sassari, Italy; 3 University of Sassari, Desertification Research Centre, Via de Nicola, I-07100 Sassari, Italy

**Keywords:** Electrical and electronic engineering, Biodiversity, Community ecology

## Abstract

This data descriptor presents a novel dataset collected using the quadrupedal robot ANYmal C in the Mediterranean coastal dune environment of the European Union (EU) habitats 2110 and 2120 in Sardinia, Italy. The dataset mainly consists of photos, videos, and point clouds of the coastal dunes, providing valuable information on the structure and composition of this habitat. The data was collected by a team of robotic engineers and plant scientists as result of a joint effort towards robotic habitat monitoring. The dataset is publicly available through Zenodo and can be used by researchers working in both the fields of robotics and habitat ecology and conservation. The availability of this dataset has the potential to inform future research and conservation efforts in the EU habitats 2110 and 2120, and it highlights the importance of interdisciplinary collaboration in the field of habitat monitoring. This paper serves as a comprehensive description of the dataset and the methods used to collect it, making it a valuable resource for the scientific community.

## Background & Summary

The European Council’s Directive 92/43/EEC (Habitats Directive)^1^ requires monitoring habitats and species of community interest listed in Annexes I and II based on specific protocols. Based on this Directive, an extensive system of protected areas, the Natura 2000 Network, was also established. Effective monitoring of the habitats, particularly inside this Network, is crucial for their conservation in the face of increasing human impact on natural environments^2–4^. Nevertheless, monitoring is a complex task requiring experience, knowledge and specific skills^5^. Nowadays, human operators are the only ones able to apply the protocols. Artificial robotics represents a new challenge for repetitive and time-consuming activities characteristic of habitat monitoring. This paper presents a new dataset related to robotic monitoring of the Mediterranean coastal dune as defined in the Habitat Directive^1^. According to the Annex I of the Directive, habitats are identified by a four-digit codex. The initial digit broadly signifies the type of environment, which, in this context, is the coastal sand dunes and inland dunes habitat. The subsequent digits provide more specificity, 2110 corresponds to Embryonic shifting dunes and 2120 to Shifting dunes with *Ammophila arenaria* (white dunes). Steep ecological gradients characterize these habitats which turn on a delicate balance between sand erosion and deposit^6,7^. The zonation of plant communities across the gradients depends on species tolerance and adaptation to dune morphology. Specifically, the two monitored habitats are located on the active dunes where dune-building grasses work to build and maintain the entire system using their roots as trap sand.

These habitats are facing a multitude of threats at a global, European and national level^8–10^. The main issues are land use change and landscape fragmentation, mechanical disturbances, trampling, invasive species, sea-level rise and extreme meteorological events. Effective monitoring at the local scale relies on selected species, including typical species (TS)^11^, which can reflect a favourable status of the structure and function of the habitat and on species having a negative role, such as alien invasive species^12,13^. The standard data collection is based on field sampling in plots (1 m × 1 m) along transects^5,14^. TS and aliens are identified inside each plot, and the cover is estimated.

**Fig. 1:**
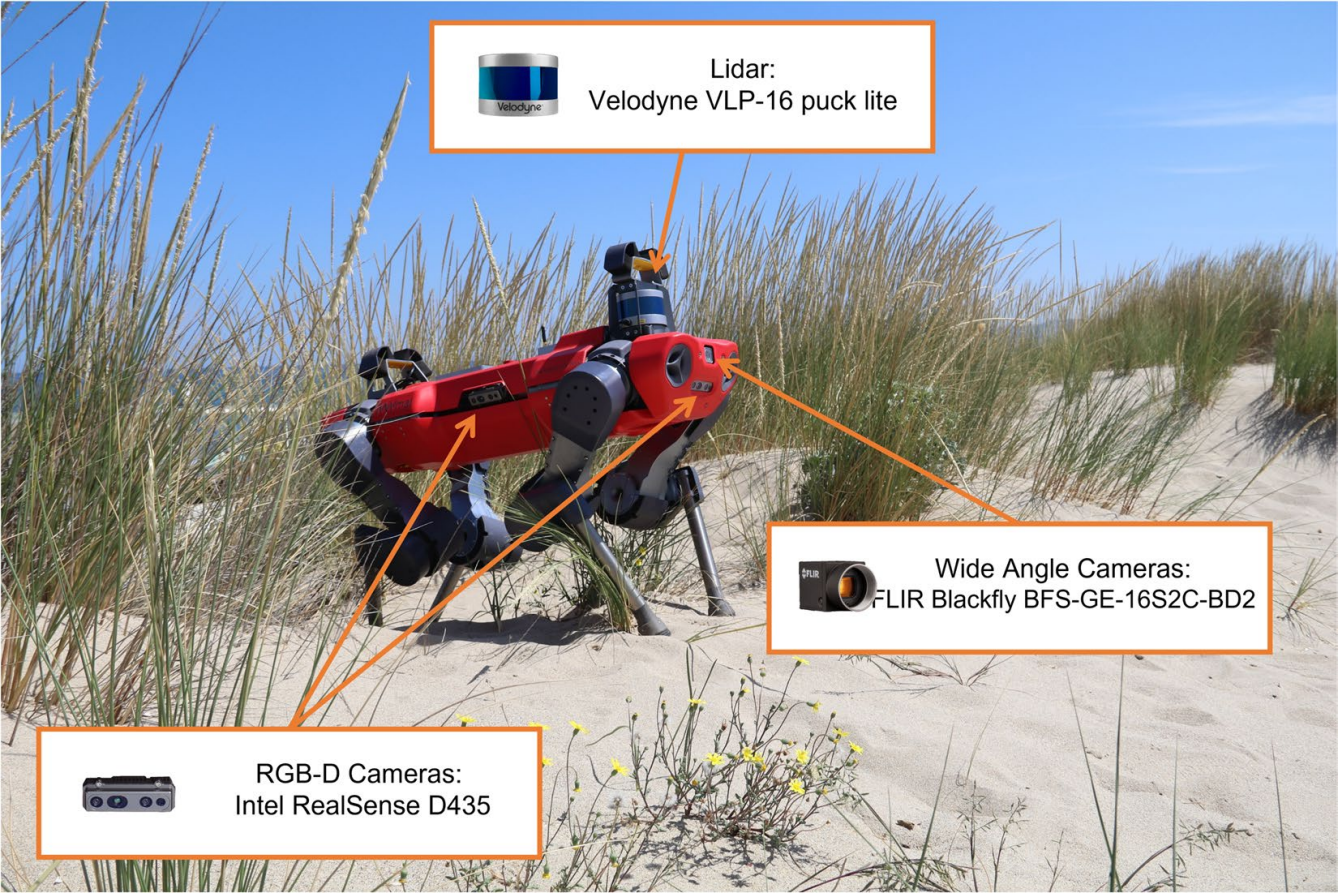
The robot ANYmal C in habitat 2120 with heighlited the sensors used for acquiring data. These sensors are one Velodyne VLP-16 puck lite, two FLIR Blackfly BFS-GE-16S2C-BD2 wide angle cameras, and four Intel RealSense D435 RGB-D cameras.

The presented data were acquired using the quadrupedal robot ANYmal C^15^ (Fig. 1), which was equipped with cameras and sensors to collect photos and videos of the dune environment. The use of robotic technology in habitat monitoring offers several advantages, including the ability to collect data from remote and inaccessible locations, and the potential for collecting data at a higher temporal resolution.

**Fig. 2:**
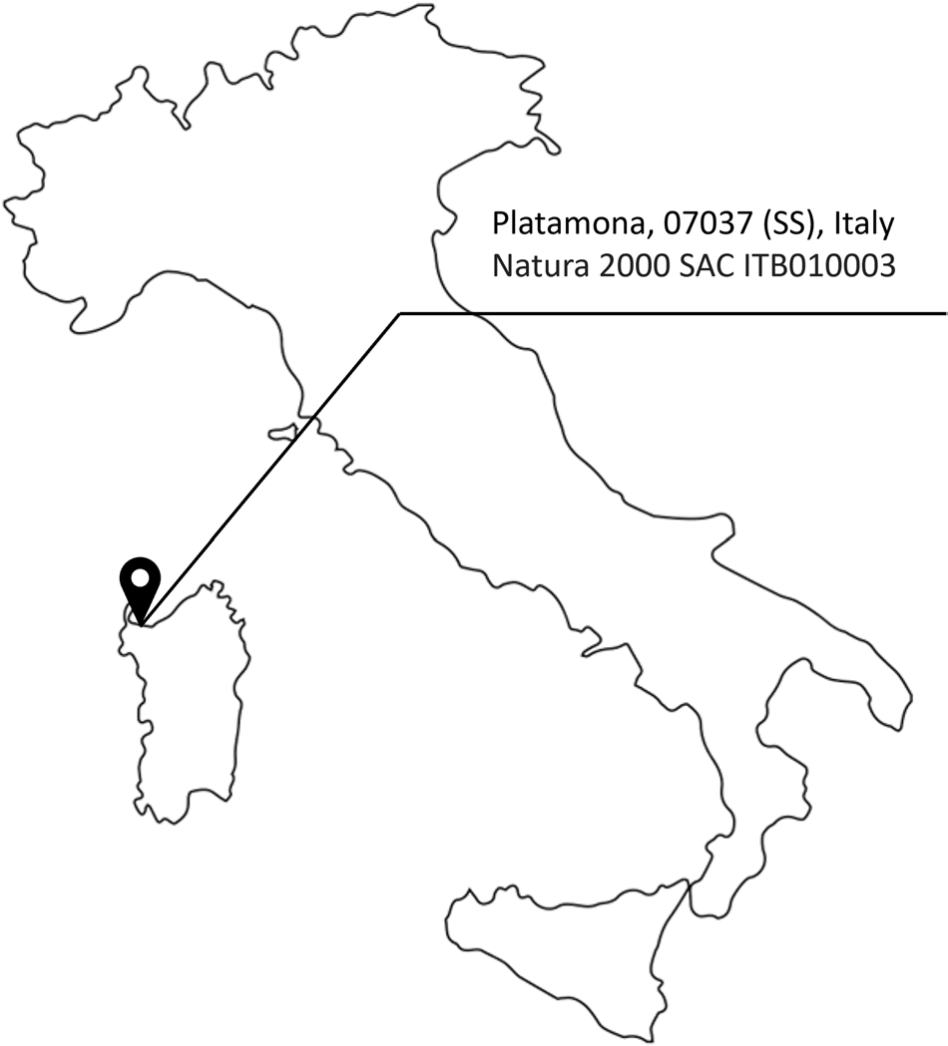
Data gathering location.

This data descriptor presents a new dataset related to robotic monitoring of the Mediterranean coastal dune environment of the EU habitats 2110 and 2120. The data were jointly collected in Sardinia, Italy (Fig. 2), by plant scientists and roboticists using the ANYmal C robot and are made publicly available through a repository^16^. Similarly to the dataset acquired in other habitats^17–19^, this data descriptor has the objective of improving current monitoring approaches by providing to researchers from different sectors the material to design, train, and test their algorithms. The combination of expertise coming from robotic engineers and plant scientists highlights the importance of multidisciplinary collaboration in habitat monitoring and conservation.

## Methods

The data used in this study were collected in Platamona, Sardinia, Italy. The study area falls within the Natura 2000 network, Special Areas of Conservation (SAC) ITB010003 - Stagno e Ginepreto di Platamona (Fig. 2). The field work was carried out over four days, from the 16th to the 19th of May 2022. Indeed this is the best period for sampling dune vegetation^5^.

The data were gathered using the robot ANYmal C (Fig. 1), developed by ANYbotics AG. The robot is capable of collecting habitat information using its onboard sensors while moving in an autonomous or teleoperated manner. ANYmal C is equipped with four Intel RealSense D435 RGB-D cameras (https://www.intelrealsense.com/depth-camera-d435/) that are placed one per side of the robot body. These cameras can register high-resolution images and videos at 30 fps. Two wide-angle FLIR Blackfly BFS-GE-16S2C-BD2 cameras are also present, one on the front and one on the back of the system. In addition, the robot mounts a Velodyne VLP-16 puck lite LiDAR (https://velodynelidar.com/products/puck-lite/) on the rear-top part of its body, which allows for the acquisition of a 3D map of the environment. The state of the robot during data acquisition was recorded as ROS bag files using the robot’s dedicated ROS interface, as per the official ROS documentation (http://wiki.ros.org/rosbag).

Before executing the field data collection, we identified suitable locations using a Geographical Information System (GIS) environment and a detailed habitat map. Thanks to this information and previous knowledge about the area, the botanic team selected for data acquisition two suitable sample areas within the SAC with an habitat coverage of at least 70%. This selection took into account also the accessibility for both the robot and the human team.

The dataset provided in this data descriptor consists of three sets of data: 3D mapping data, species data, and monitoring mission data. Details on the acquisition methods of these data sets are provided in the subsequent sections of this paper.

### Species data

We collected data for four indicator species, including the TSs of the two habitats^5^, namely *Calamagrostis arenaria* (L.) Roth (habitat 2120) and *Thinopyrum junceum* (L.) Á.Löve and *Achillea maritima* (L.) Ehrend. & Y.P.Guo (habitat 2110) and one alien invasive. We refer to World Flora Online Portal (http://www.worldfloraonline.org) for the nomenclature, but Table 1 reports also the common name of these indicator species for the convenience of non-plant-expert readers. *T. junceum* sand couch-grass, and *A. maritima*, cottonweed, are salt-tolerant species living at the base of the dunesands. *C. arenaria*, European beach grass, is a perennial plant up to 120 cm tall with horizontal and vertical rhizomes. It is a keystone species crucial in dune construction and maintenance. Concerning the alien invasive species, we collected data for *Carpobrotus acinaciformis* (L.) L.Bolus. This species, native to South Africa, was introduced in Europe in the 1900s, becoming the most abundant invasive species in dune habitats^20^. The impact of this species on coastal habitats is well documented^20,21^. For each indicator species, at least 130 pictures and 13 videos were recorded using the RGB-D front camera. In the case of *C. arenaria*, 160 more images and 16 more videos were recorded using the front wide camera due to the species size. Table 1 reports the number of photos and videos provided in the present dataset.

**Table 1:** The three species typical of the habitats 2110 and 2120 and the alien species with the number of photos and videos for each of them.

Scientific Name	Common Name	Type	# Photos	# Videos
*Calamagrostis arenaria* (L.) Roth	Marram grass	Habitat 2120 typical species	348	35
*Achillea maritima* (L.) Ehrend. & Y.P.Guo	Cottonweed	Habitat 2110 typical species	160	16
*Thinopyrum junceum* (L.) Á.Löve	Sand couch-grass	Habitat 2110 typical species	130	13
*Carpobrotus acinaciformis* (L.) L.Bolus	Hottentot figs	Alien species	160	16

All data are captured usign the depth camera, except for *C. arenaria* pitures and videos, which are captured in part with the depth camera and in part with the wide camera.

**Fig. 3:**
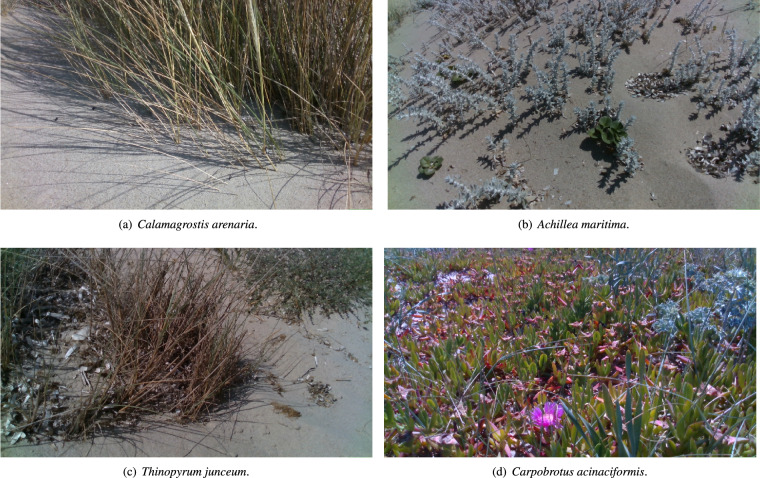
The four indicator species of habitats 2110 and 2120. These pictures were taken by the on-board depth cameras of the robot. (**a**–**c**) are typical species. (**d**) is an alien species.

The process of data acquisition involved the following steps: firstly, the plant experts followed the instructions provided in^22^ to recognize the indicator species mentioned earlier. Following this, a robotic specialist remotely operated the robot and directed it towards the identified instance of the species. Due to this procedure, each photograph of a specific species necessarily includes at least one instance of it, but also other indicator species may be present. Figure 3 presents an illustration for each of the four species discussed previously.

### 3D Mapping data

The second section of the dataset consists of 3D spatial data obtained through a LiDAR sensor and shared in the form of point clouds. These point clouds provide information on the dimensions and shapes of the surrounding environment. The acquisition of this set of data is performed while the robot is guided by an operator. First, the robot is placed in selected study area, and GPS data, date, and weather information are annotated to enable data comparison with past and future surveys. The employed sensor was GPS Garmin Montana 680, and the measurements were taken with at least 3 m accuracy. The geographic coordinate system is EPSG 4326. Next, the mapping process begins through the ANYmal’s ROS Graphical User Interface, and ANYmal is directed by the operator to move forward, execute a 180° turn, and return to its starting position. The point cloud is stored at the end of this procedure.

Mapping data of five different areas in habitats 2110 and 2120 are presented through this data descriptor. Table 2 provides additional information on these.

**Table 2:** Details of the mappings: date, time, weather information, and georeferencing information (EPSG: 4326).

Name	Date	Time	Weather	Latitude	Longitude
Mapping 1	17 May 2022	10:40	Sunny	40°49′15.511″N	8°30′8.691″E
Mapping 2	17 May 2022	14:30	Sunny	40°49′15.768″N	8°30′6.969″E
Mapping 3	17 May 2022	14:52	Sunny	40°49′15.225″N	8°30′6.976″E
Mapping 4	17 May 2022	16:47	Sunny	40°49′14.783″N	8°30′8.753″E
Mapping 5	19 May 2022	10:21	Sunny	40°49′15.512″N	8°30′10.821″E

### Monitoring mission data

The final section of the dataset pertains to the autonomous monitoring mission carried out by the robot in Platamona in the same plots surveyed by plant scientists. GPS coordinates (EPSG 4326) were recorded to enable comparisons with past and future surveys. Weather information was also recorded. This could be employed to estimate sunlight.

**Fig. 4:**
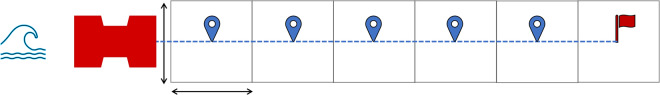
Example of robot motion during the autonomous monitoring mission: 6 m transect. The robot starts pointing towards the opposite direction of the sea, and it moves on a straight line (dashed blue path) till the final goal (red flag). At each waypoint (blue marks) the robot stops. For the entire mission, the RGB-D cameras record a video, the robot state is stored as ROS bag files. Shorter and longer transects can be performed changing the number of waypoints.

For the autonomous monitoring, a 3D map of the environment obtained through the mapping process described in the Section “3D Mapping Data” was used to assist the robot in localizing itself within the environment. The robot followed a straight path going in the direction from the shoreline to the hinterland, as shown in Fig. 4. At each meter, the robot stopped, and four photos (one per each of the four RGB-D cameras) were taken, along with videos recorded by the same cameras, and ROS bags (http://wiki.ros.org/rosbag) of robot status information. A total of six autonomous plots have been performed. More information regarding them can be found in Table 3. This table specifies also the mapping associated to each plot. Finally, Table 4 lists the ROS topics used to store the robot status information. This includes the robot’s position, velocity and battery charge, and the joint positions, velocities, accelerations, torques and currents.

**Table 3:** Details of the six monitoring missions.

Name	Point Cloud	Length	Date	Time	Weather	Latitude	Longitude
Plot 1	Mapping 1	12 m	17 May 2022	10:46	Sunny	40°49′15.511″N	8°30′8.691″E
Plot 2	Mapping 2	18 m	17 May 2022	14:41	Sunny	40°49′15.768″N	8°30′6.969″E
Plot 3	Mapping 3	20 m	17 May 2022	14:57	Sunny	40°49′15.225″N	8°30′6.976″E
Plot 4	Mapping 3	10 m	17 May 2022	15:03	Sunny	40°49′14.513″N	8°30′6.945″E
Plot 5	Mapping 4	25 m	17 May 2022	16:52	Sunny	40°49′14.783″N	8°30′8.753″E
Plot 6	Mapping 4	20 m	17 May 2022	17:00	Sunny	40°49′14.793″N	8°30′8.917″E

The table specifies the point cloud used to enable the autonomous locomotion, the transect length, the date, time, weather information, and georeferencing information (EPSG: 4326).

**Table 4:** List of ROS topics from which we recorded the robot status during mapping and autonomous missions.

Topic Name	Description	M	A
/state_estimator/anymal_state	Robot info, e.g., base position [m] and orientation [rad], joint position [rad], velocity [rad/s], acceleration [rad/s^2^], and torque [Nm].	✓	✓
/log/state/current/LF_HAA	Current [A] of the hip adduction/abduction joint of the left fore leg	✓	✓
/log/state/current/LF_HFE	Current [A] of the hip flexion/extension joint of the left fore leg	✓	✓
/log/state/current/LF_KFE	Current [A] of the knee flexion/extension joint of the left fore leg	✓	✓
/log/state/current/LH_HAA	Current [A] of the hip adduction/abduction joint of the left hind leg	✓	✓
/log/state/current/LH_HFE	Current [A] of the hip flexion/extension joint of the left hind leg	✓	✓
/log/state/current/LH_KFE	Current [A] of the knee flexion/extension joint of the left hind leg	✓	✓
/log/state/current/RF_HAA	Current [A] of the hip adduction/abduction joint of the right fore leg	✓	✓
/log/state/current/RF_HFE	Current [A] of the hip flexion/extension joint of the right fore leg	✓	✓
/log/state/current/RF_KFE	Current [A] of the knee flexion/extension joint of the right fore leg	✓	✓
/log/state/current/RH_HAA	Current [A] of the hip adduction/abduction joint of the right hind leg	✓	✓
/log/state/current/RH_HFE	Current [A] of the hip flexion/extension joint of the right hind leg	✓	✓
/log/state/current/RH_KFE	Current [A] of the knee flexion/extension joint of the right hind leg	✓	✓
/pdb/battery_state_ros	Battery information, e.g., charge percentage, voltage [V], current [A].	✓	✓
/tf	Coordinate frames and transformations between them (TFs) (http://wiki.ros.org/tf2)	✓	✓
/path_planning_and_following/navigate_to_goal/result	Info on the success (or failure) of the robot in reaching the desired navigation goal	✗	✓
/path_planning_and_following/trajectory_poses	The planned Cartesian poses (waypoints) tracked by the robot to reach the goal	✗	✓
/path_planning_and_following/active_path	The actual Cartesian path through the waypoints followed by the robot to reach the goal	✗	✓

The columns **M** and **A** indicate which topic has been saved during mapping and an autonomous mission, respectively.

## Data Records

The data are provided through^16^ at 10.5281/0.8314728. An example code to use the data saved in the ROS bag files is also on GitHub (https://github.com/mpollayil/Code-for-Habitat-Data-Analysis) and on^23^.

**Fig. 5:**
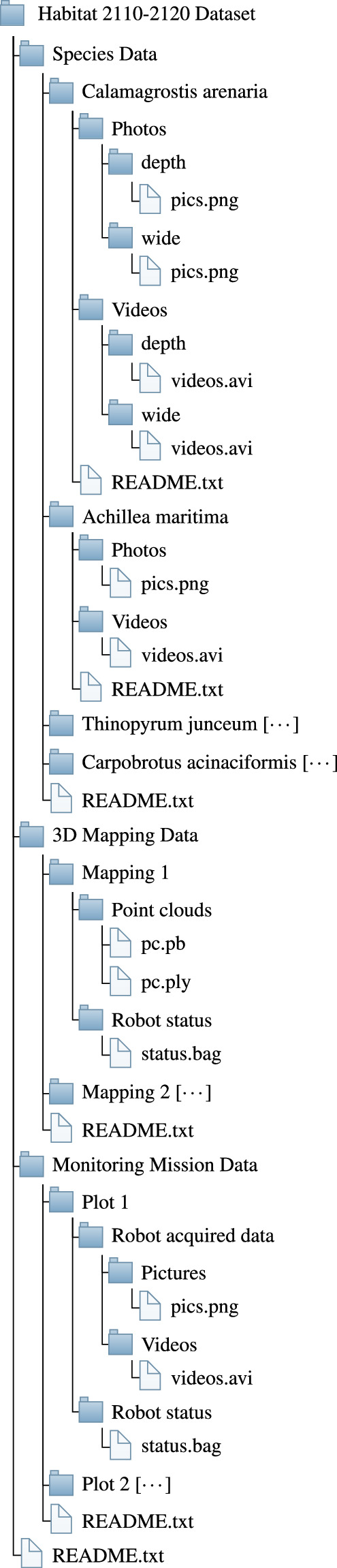
Hierarchical directory structure of the dataset. The symbol [⋯] specifies that the content structure of that folder is similar to the other same-level folders.

We provide three sets of structured data, represented in the tree diagram in Fig. 5. Each subtree includes a README.txt file that describes the corresponding data set. The set “Species Data” includes pictures of the four indicator species found in habitats 2110 and 2120. Each subfolder named after the indicator species contains a “Photos” and a “Videos” subfolder containing the relevant files. In the case of *Calamagrostis arenaria*, an additional directory layer is present to distinguish between data acquired with the RGB-D camera, i.e. “depth” folder, and with the wide camera, i.e., “wide” folder Table 1 displays the number of pictures for each indicator species.

The set “3D Mapping Data” comprises five Mapping subfolders, each containing point clouds in the “Point clouds” folder and the stored robot status in the “Robot Status” folder.

The final set is “Monitoring Mission Data”, which includes all the information gathered during autonomous monitoring missions executed by the robot. This set is divided into six subfolders for the six plots, each one including the robot status and acquired data.

### Data formats

This section provides technical details about the data file formats.

#### .jpg

Images acquired by the robot are saved in the jpg format, a prevalent image standard that can be accessed using widely accessible image viewers across different operating systems. Both species pictures and photos recorded during monitoring missions are in this format.

#### .avi

Videos registered with the robot’s on-board cameras are stored in avi format, and they can be be accessed using widely accessible video players across different operating systems.

#### .ply and .pb

Three-dimensional mappings are saved as point clodus and provided to users in two standards formats: ply and pb. Ply is the Polygon File Format, a common file extension for 3D models that can be accessed through common software such 3D modeling tools, e.g., MeshLab, or math processing products, e.g., MATLAB (https://www.mathworks.com/products/matlab.html). Pb is the most widely used point cloud format within ROS, particularly in ANYmal research (https://www.anymal-research.org/).

#### .bag

**Fig. 6:**
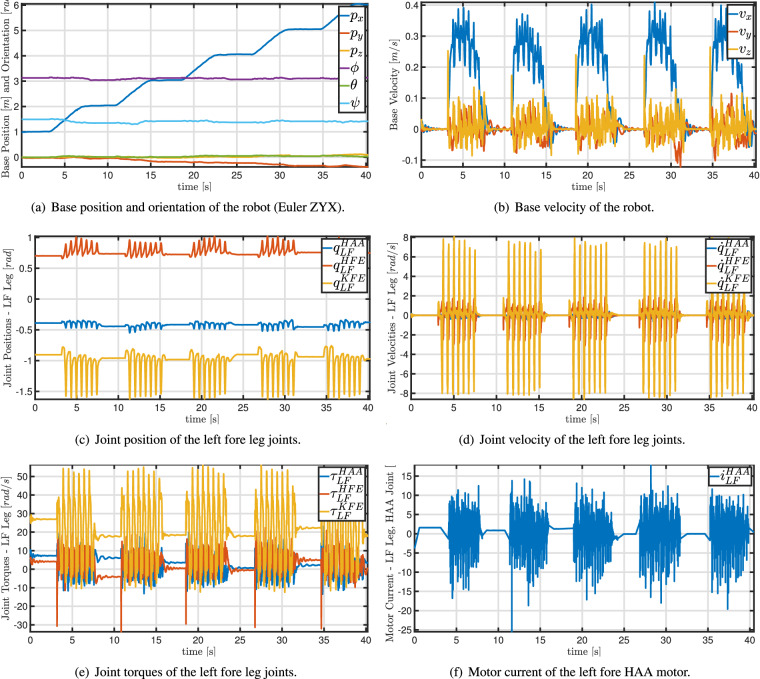
Graphs of the robot status extracted from the ROS bag of the Plot5. The robot moved as shown in Fig. 4. For the sake of readibility, only the first 40 s have been plotted.

ROS employs topics (http://wiki.ros.org/rostopic) to transmit the robot information and data. Data flowing in ROS topics can be stored and shared as bag files. Table 4 summarizes the topics included in the provided bag files. More details about these topics can be found on the ANYmal Research site (https://www.anymal-research.org/). Figure 6 displays some of measured quantities during the monitoring mission Plot5. Within ROS, there exist various tools for extracting, visualizing, and analyzing the data saved in bag files. MATLAB (https://www.mathworks.com/products/matlab.html) can also be a valid tool for researchers outside of robotics to handle this file type.

### Template script for data extraction

In Code 1, we show a template example of a MATLAB script for extracting data from ROS bags and reading the content of a specific ROS topic. The ROS toolbox and MATLAB 2022a (https://www.mathworks.com/products/matlab.html) or later versions can be used to run this example. Table 4 reports the topics that can be extracted from the bags. The ANYmal Research Website (https://www.anymal-research.org/) describes these issues in detail, as well as the specifics of the sensors mounted on the robot. To access this, however, a research collaboration with ANYbotics is required.

#### Code 1.

Example of MATLAB script to extract data from ROS bag files.

We must emphasize that being part of ANYmal Research is not required to access the data offered in this research. Also, script displayed in Code 1 and the code provided by this data descriptor are sufficient to extract, visualize, and analyze the supplied data.

## Technical Validation

During the fieldwork, the robotics engineering team — Franco Angelini (FA), Mathew Jose Pollayil (MJP), and Manolo Garabini (MG) — and the plant science team — Simonetta Bagella (SB), Maria Carmela Caria (MCC), and Giovanni Rivieccio (GV) — both offered quality assurance. Each author kept tabs on the data gathering process and thoroughly examined the final dataset for mistakes and inconsistencies.

It is worth mentioning that that the data are presented in their raw state, without any post-processing that could jeopardize their authenticity. The next section discusses a few choices that guarantee the validity of the data acquisition.

### Area selection

The sampling sites have been chosen by the team of plant scientists, who are expert on the local flora and plants. Platamona, located in the north-west of Sardinia, in the centre of the Gulf of Asinara, is a shoreline made of fine sand. It is the longest and deepest beach along the Sardinian coastline: about 15 km long and 10 to 30 m wide. The two habitats 2110-Embryonic shifting dunes and 2120-Shifting dunes with *Ammophila arenaria* (white dunes) occupy large surfaces in the dunal system.

### Time selection

The survey time, 16th–19th of May 2022, established by the plant scientists, was the most suitable for the quick identification of the species to be monitored based on the presence of the flowers and the optimal development of vegetative apparatus.

### Species data validity

TS for habitats 2110 and 2120 have been selected in accordance with EU guidelines^5^. Plant scientists SB, MCC and GR identified them in the fields and in laboratory using the Flora Manual^22^ ensuring the correct species classification.

### Mapping data validity

The data that the robot saved during mapping is presented in its unprocessed raw form. Since ANYmal uses the stored map to locate itself in the environment using the Simultaneous Localization and Mapping (SLAM) algorithm, its authenticity is confirmed by the lack of any localization errors communicated by the robot during this operation.

### Monitoring data validity

Scientific journals^24^ has been used to drive the procedures employed during monitoring missions, assuring the authenticity of the data gathered during them. To follow these rules, the robot imitates field relevé work done by plant biologists. Additionally, the robot is programmed to alert users in case of potential data acquisition errors. The data are supplied for analysis without any alterations, therefore there is no chance of post-processing-related corruption.

### Database validity

After the data collection was finished, the database was created. We only included accurate, clean data. Each database entry was thoroughly reviewed by both teams to assure its accuracy. The dataset’s photos, videos, and classification in the “Species Data” section were all confirmed by the plant specialists. Similar to this, the robotic engineering team verified the accuracy of the point clouds and robot status information by reviewing them and executing test scripts.

## Usage Notes

The provided data has a multidisciplinary scope and can benefit both robotic and botanical research. Data contained in the “Species Data” section can be employed by computer scientists to test or train machine learning techniques to detect and classify flora, similarly to^25,26^. Engineers could also employ point cloud and robot status information contained in the “3D Mapping Data” section to validate their navigation or locomotion algorithms. Finally, the section “Monitoring Mission Data” contains georeferenced information regarding plots that could be used by computer scientists to validate their plant species classification algorithms or by plant scientists to evaluate the state of habitat conservation and to compare them to other plots, such as historical or future data from the same locations.

## Data Availability

The MATLAB code that is associated with the data provided in this paper is hosted on the GitHub pages of the Research Center E. Piaggio (https://github.com/mpollayil/Code-for-Habitat-Data-Analysis) and on^23^. Data from ROS bag files may be extracted and visualized with this. The GitHub repository, in particular, has a README file that details every script separately.
